# Effects of high oleic acid peanuts on mice’s liver and adipose tissue metabolic parameters and gut microbiota composition

**DOI:** 10.3389/fnut.2023.1205377

**Published:** 2023-07-27

**Authors:** Sarit Anavi-Cohen, Nina Tsybina-Shimshilashvili, Gil Zandani, Ran Hovav, Noa Sela, Abraham Nyska, Zecharia Madar

**Affiliations:** ^1^Peres Academic Center, Rehovot, Israel; ^2^The Faculty of Agriculture, Food and Environment, The Hebrew University of Jerusalem, Rehovot, Israel; ^3^Department of Field Crops and Vegetables Research, Plant Sciences Institute, Agricultural Research Organization, Rishon LeZion, Israel; ^4^Department of Plant Pathology and Weed Research, Volcani Center, Rishon LeZion, Israel; ^5^Sackler School of Medicine, Tel Aviv University, Tel Aviv, Israel

**Keywords:** peanuts, oleic acid, liver, adipose-tissue, gut microbiota

## Abstract

This study aimed to investigate the effects of two types of peanuts, regular Hanoch (HN) and a new high-oleic cultivar., Hanoch-Oleic (HO), on metabolic parameters and gut microbiota composition. Male C57BL/6 mice were fed with a normal diet (ND) or ND supplemented with HN (NDh) or HO (NDo). Following 18 weeks of diet regimen, the NDo group exhibited reduced body weight and peri-gonadal adipose-to-body weight ratio, paralleled to lesser food consumption. Although blood levels of total cholesterol, HDL-cholesterol, free fatty acids, and liver enzyme levels did not differ between groups, decreased insulin sensitivity was found in the NDh group. Within adipose tissue, the expression of lipolytic and lipogenic enzymes was higher, while those related to lipid oxidation were lower in the NDh group compared to the NDo group. Additionally, HO peanuts consumption promoted the establishment of a healthy microbiota, with an enhanced abundance of *Bifidobacterium, Lactobacillus*, and *Coprococcus* genera. In conclusion, the inclusion of the HO peanut cultivar., rather than the conventional peanut cultivar., in a balanced diet was related to better metabolic outcomes and was linked to a favorable microbiota profile.

## Introduction

Peanuts (*Arachis hypogaea*) are legumes from a botanic perspective. Yet, they are nutritionally classified as nuts and have comparable culinary use (1). Peanuts are a rich source of macro and micronutrients, as well as phytochemicals, such as polyphenols, isoflavones, phytosterols, and resveratrol (2, 3). Regarding their macronutrient composition, fats constitute the dominant part. Unsaturated fatty acids make up about 80% of all fats, with 50% of them consisting of monounsaturated fatty acids (MUFA) and 30% polyunsaturated fatty acids (PUFA). Saturated fat contents in peanuts are low, whereas trans fats and cholesterol are completely absent (4, 5). Among MUFA, oleic acid is considered health-promoting with much impact on all body systems (6). For example, in Type 2 diabetes patients, oleic acid was found to diminish inflammation and oxidative stress and to improve glucose homeostasis, as manifested by the amelioration of insulin resistance, and increased pancreatic beta-cell survival (6–8). Recently, a high-oleic Virginia-type peanut cultivar., known as “Hanoch-Oleic” (HO) was developed in Israel. Compared to the leading nowadays variety “Hanoch” (HN), this new strain consists of higher oleic acid contents while the overall fat fraction remained similar. Specifically, the HO fat fraction is composed of 82.7% oleic acid (vs. 55% in HN), 2% linoleic acid (vs. 25% in HN), 4.9% palmitic acid, and ~10% of other fatty acids. More favorable health effects are anticipated as a result of the increase in oleic acid and/or the decrease in linoleic acid.

The impact of regular intake of peanuts on diverse diseases and pathologic conditions has been recently reviewed (1). Notwithstanding inconsistencies, data obtained from epidemiological and clinical investigations advocate the health-promoting outcomes of peanuts inclusion in the diet. Most of the research that has been conducted to date specifically with peanuts, used the regular, in terms of fatty-acids composition type. Given the recognized importance of different types of fatty acids on multiple systems, it is highly imperative to explicitly evaluate the consequences induced by high-oleic peanuts.

An essential factor influencing the host’s health is the gut microbiota (9). Changes in the composition of the gut microbiome are directly related to dietary content and the nutrients taken by the individual. A dysbiosis state brought on by an unhealthful, imbalanced diet can encourage the onset and/or the progression of several pathological illnesses/conditions, including obesity, nonalcoholic fatty liver disease (NAFLD), inflammation, inflammatory bowel disease (IBD), and cancer (10, 11).

The close anatomical proximity of the liver, visceral white adipose tissue, and intestine has given rise to the concept of the “gut-adipose tissue-liver” axis which contends that these tissues interact functionally. The “gut-liver” axis includes the interaction of intestinal microbiome metabolites with liver receptors (12). Consequently, the gut microbiota may directly influence the development of hepatic inflammation and liver disorders (13). As an alternative, according to this supposition, numerous additional compounds made by gut bacteria may also reach the liver and elicit positive effects on this tissue. The existence of the “gut-adipose tissue” axis has been supported by mounting data, albeit being considerably less clearly defined. A comprehensive understanding of the effects exerted by habitual peanuts consumption on the aforementioned axes is currently lacking.

This work was conducted to elucidate the effects of two peanuts strains, namely “Hanoch” and “Hanoch-Oleic” which contain regular or augmented oleic contents, respectively, on metabolic parameters in the liver and adipose tissue, as well as on gut microbiota composition.

## Materials and methods

### Experimental animals, and diets

All animal experiments were done in accordance with the rules of ethics of the Hebrew University of Jerusalem, Israel (AG-16-14784-2) and were approved by Institutional Animal Care Ethics Committee.

Male C57BL/6 J four-week-old mice (*n* = 24), were randomly divided into 3 groups, respectively (*n* = 8 per group) and housed in cages. All mice were maintained at 22 ± 2°C, with controlled moisture of 60%, and in a 12 h light/12 h dark cycle with *ad libitum* access to food and water. After an acclimation period of standard rodent food, the food was replaced by experimental diets. Two peanut cultivars: “Hanoch” (HN, 55% oleic acid) and “Hanoch-Oleic” (HO, 80% oleic acid) were added to the normal diet (ND). The composition of the two peanut varieties is presented in Table 1.

**Table 1:** Seed composition of the two peanut varieties.

	Peanut variety	
Compounds	Hanoch (HN)	Hanoch-oleic (HO)
Carbohydrates %	20.9	21.5
Protein %	23.5	22.6
Fat %	49.5	48.7
C16:0 (palmitic acid) %	8.4	4.99
C18:0 (stearic acid) %	3.23	2.03
C18:1n9 cis (oleic acid) %	55.28	82.68
C18:2n6 (linoleic acid) %	25.01	2.08

HN, conventional peanut strain; HO, high oleic peanut strain.

Experimental groups were as follows: (1) ND, (2) ND with “Hanoch” (NDh) peanuts, w/w 4%, (3) ND with high-oleic acid “Hanoch-Oleic” (NDo) peanuts, w/w 4%. The composition of the diets is detailed in Table 2. Of note, the control, ND group, was also used as a control group in a previously published study (14). All mice were on these diets for 18 weeks until elimination. Body weight was recorded once a week and food intake was measured three times weekly.

**Table 2:** Diet consumption.

Ingredients	ND	NDh	NDo
Casein (g)	21	20.19	20.19
L-Methionine (g)	0.3	0.29	0.29
Corn Starch (g)	50	48.08	48.08
Maltodextrin (g)	10	9.62	9.62
Anhydrous milkfat (g)	2	1.92	1.92
Sucrose (g)	3.9	3.75	3.75
Cellulose (g)	3.5	3.37	3.37
Soybean oil (g)	2	1.92	1.92
Lard (g)	2	1.92	1.92
Peanut (g)	-	3.85	3.85
Mineral mix (g)	3.5	3.5	3.5
Vitamin mix (g)	1.5	1.5	1.5
Choline Chloride (g)	0.3	0.3	0.3
BHT (g)	0.014	0.014	0.014
Total (g)	100	100	100
Total (kcal)	394.89	403.6	403.28
Protein (%)	21.58	21.69	21.64
Carbohydrate (%)	64.75	62.14	62.20
Fat (%)	13.68	15.81	15.73

ND, normal diet; NDh, normal diet plus Hanoch 4% (w/w), the conventional peanut strain; NDo, normal diet plus the high oleic peanut strain Hanoch-Oleic 4% (w/w).

### Oral glucose tolerance test

On week 16, an oral glucose tolerance test (OGTT) was performed. Mice were fasted overnight (12 h) and then weighed and marked. D-glucose was dissolved in a 30% solution and administered to mice by oral gavage at doses of 10 μL per 1 g body weight. Glucose levels were measured before and 30, 60, and 120 min after the glucose loading. Blood was taken from the tail tip and glucose levels were measured using a glucometer (Free Style Optimum Neo, Oxon United Kingdom).

### Animal sacrifice and organ collection

At the end of the 18th week, 12 h-fasted mice were weighed and randomly eliminated by isoflurane USP inhalation. Blood was drawn from the vena cava. Liver and peri-gonadal adipose tissues were removed, weighed, placed in liquid nitrogen, and stored at −80°C. The cecum content was collected and stored at −80°C for microbiota analysis.

### Plasma analysis

Plasma was obtained by centrifugation at 8,000 × rpm at 4°C for 10 min and stored at −20°C. blood liver enzymes and lipid profile were measured (American Laboratory, Herzliya, Israel). Plasma insulin levels were determined using an Insulin Rat/Mouse ELISA Kit (Cat. #EZRMI – 13 K). The HOMA-IR index was calculated by using the following formula: [Fasting insulin concentration (microU/mL) * Glucose concentration (mg/dL) /405]. Free fatty acids in plasma were determined using an Abcam Free Fatty Acids Quantification Assay Kit (ab65341) according to the manufacturer’s instructions.

### Histological examination and determination of triglyceride levels in liver tissue

During mice sacrificing, a small portion of the right lobe of the liver was cut off and placed in formaldehyde 4%. Histological slides were prepared by Patholab (Rehovot, Israel) and examined as previously described (15). Briefly, liver samples were dissected, placed in plastic cassettes, and subjected to a dehydration process. Following dehydration, the samples underwent embedding in paraffin blocks using an automated apparatus. Subsequently, serial sections, approximately 3–5 μm thick, were cut from each block and transferred onto glass slides. These sections were then stained with hematoxylin and eosin (H&E) and covered using an automated apparatus. Histopathological changes were assessed and evaluated by the study pathologist. A semiquantitative grading system consisting of five grades (0–4) was employed, considering the severity of the observed changes. The assigned scores indicate the dominant degree of the specific lesions observed throughout the entire field of the histology section. The grading criteria utilized were as follows: zero (0) indicates the absence of lesions; 1 signifies minimal change; 2 represents mild change; 3 indicates moderate change; and 4 denotes marked change.

Liver triglyceride levels were measured by the Triglyceride Quantification Assay kit (Abcam, ab-65,336) according to the manufacturer’s instructions.

### Protein extraction and Western blotting

Total protein was extracted from liver and adipose tissue with a lysis buffer containing: 20 mM Tris–HCl (pH 7.4), 145 mM NaCl, 10% glycerol, 5 mM EDTA, 1% Triton X-100, 0.5% NP-40, 100 μM PMSF, 200 μM NaVO4, 5 mM NaF, and 1% protease inhibitor cocktail. To remove protein, 50 mg of liver or 100 mg of adipose tissue respectively, was weighed and homogenized with 500 μL lysis buffer. Lysates were centrifuged at 14,000 × rpm at 4°C for 15 min twice, and the protein concentration was determined by the Bradford method with 200 μM BSA used as a standard. Then, 40 μg of each sample was electrophoresed through 10% SDS-PAGE, after which proteins were transferred onto 0.2 μm nitrocellulose membranes. To inhibit non-specific proteins on transferred membranes, blocking was completed using a 5% skim milk solution (BD Difco skim milk) diluted in TBST × 1. Blots were incubated with primary antibodies:anti-rabbit AMPK diluted 1:10,000, pAMPK diluted 1:10,000 (Thr-172), anti-rabbit ACC diluted 1:1,000, pACC diluted 1:10,000 (Cell Signaling Technology, Beverly, MA, United States); anti-mouse β-actin diluted 1:5,000 and anti-mouse iNOS diluted 1:1,000 (BD biosciences, United States); anti-rabbit CREB diluted 1:1,000, pCREB diluted 1:1,000 and anti-rabbit CD36 diluted 1:1,000 (Abcam, United Kingdom). Then, after several washes, blots were incubated with secondary goat antibodies diluted 1:10,000 (Jackson Immuno Research Laboratories, West Grove, PA, United States). The immune reaction was detected by enhanced chemiluminescence, with bands being quantified by densitometry and expressed as arbitrary units. An unspecific band out of the total protein (Ponceau) was used as a housekeeping protein.

### Quantitative real-time polymerase chain reaction

Total RNA was isolated from liver tissue by using the Tri-Reagent (Sigma-Aldrich, United States) method and from the adipose tissue by using the RNeasy Lipid Tissue Mini Kit, Qiagen (Cat. 74804). cDNA was prepared by reverse transcription by a High-Capacity cDNA Reverse Transcription Kit (Quanta BioSciences, Gaithersburg, MD, United States). Quantitative real-time PCR (RT-qPCR) was performed using the 7300 Real-Time PCR System (Applied Biosystems, Foster City, CA, United States). Quantitative changes in gene expression were normalized by 18S mRNA as a reference gene. The primer sequences and probes used for RT-qPCR are described in Table 3.

**Table 3:** Primers sequences.

Name	Reverse	Forward
18 s	5′-CCTCAGTTCCGAAAACCAAC-3′	5′-ACCGCAGCTAGGAATAATGG-3′
Fasn	5′-GGTCGTTTCTCCATTAAATTCTCAT-3′	5′-CTAGAAACTTTCCCAGAAATCTTCC-3′
SREBP-1C	5′-TAGATGGTGGCTGCTGAGTG-3′	5′-GATCAAAGAGGAGCCAGTGC-3′
TNFα	5′-CCACAAGCAGGAATGAGAAGA-3′	5′-ACGTGGAACTGGCAGAAGAG-3′
HSL	5′-TGCCCAGGAGTGTGTCTGAG-3′	5′-AGGACACCTTGGCTTGAGCG-3′
ATGL	5′-GGTTCAGTAGGCCATTCCTC-3′	5′-GTGCAAACAGGGCTACAGAG-3′
CPT-1	5′-CAGCGAGTAGCGCATAGTCA-3′	5′-TGAGTGGCGTCCTCTTTGG-3′
IL-6	5′-TGCAAGTGCATCATCGTTGT-3′	5′-ACTTCACAAGTCGGAGGCTTAAT-3′
PPARγ	5′-CAGCTTCTCCTTCTCGGCCT-3′	5′-CACAATGCCATCAGGTTTGG-3′
PGC-1α	5′-AGAGCAAGAAGGCGACACAT-3′	5’-AACAAGCACTTCGGTCATCC-3′
PPARα	5′-CTGCGCATGCTCCGTG-3′	5’-CTTCCCAAAGCTCCTTCAAAAA-3′
G6Pase	5′-AAGAGATGCAGGAGGACCAA-3′	5’ACTCCAGCATGTACCGGAAG-3′
PEPCK	5′-TGCAGGCACTTGATGAACTC-3′	5’-CAAACCCTGCCATTGTTAAG-3′

18S, 18S ribosomal RNA; Fasn, Fatty acid synthase gene; SREBP-1C, Sterol regulatory element-binding transcription factor 1; TNFα, Tumor necrosis factor alpha; HSL, Hormone sensitive lipase; ATGl, Adipose triglyceride lipase; CPT-1, Carnitine palmitoyl transferase I; IL-6, Interleukin 6; PPARγ, Peroxisome proliferator-activated receptor gamma; PGC-1 α, Peroxisome proliferator-activated receptor gamma coactivator 1-alpha; PPAR α, Peroxisome proliferator-activated receptor alpha; G6Pase, Glucose 6-phosphatase; PEPCK, Phosphoenolpyruvate carboxykinase; AdipoR1, Adiponectin receptor 1; AdipoR2, Adiponectin receptor 2; SAA-1, Serum amyloid A1.

### Metagenomics

#### Preparation of 16S ribosomal RNA gene amplicons for the Illumina system

The effects of each diet on the bacterial population in the gut microbiome were examined with the analysis of the prokaryotic 16S ribosomal RNA gene (16S rRNA), using a two-step PCR-based method for preparing samples for sequencing the variable V3 and V4 regions of the 16S rRNA gene. 16S paired-end amplicon sequences were demultiplexed, preprocessed, and analyzed using QIIME2 (v2019.07) (16) and the DADA2 denoising pipeline (17). Sequences with 97% similarity were assigned to the same operational taxonomic units (OTU). OTUs were taxonomically assigned using qiime2 q2-feature-classifier with greengenes version 13_8 (18). OTUs relative abundances were used to calculate and analyze rarefaction curves. Bacterial richness and diversity within samples were classified by alpha diversity (Pielou’s index, observed-species indices, and Shannon index). linear discriminant analysis (LDA) effect size (LEfSe) was calculated and visualized using LEfSe available as a Galaxy module^1^ (19). Functional analysis of the microbiome was done using PICRUSt2 (20) with QIIME2 plugin. Differential abundance analysis was done with DESeq2 (21). Visualization of PCA and Heatmap of differentially abundant functionality was done using the R package Clustvis (22).

### Statistical analysis

The results are presented as means ± standard error (SEM). For all analyses, the JMP 14 Pro Software Suites (SAS Institute, Cary, NC, United States) was used. Comparisons between groups were made by one-way analysis of variance (ANOVA), followed by a Tukey–Kramer test or by unpaired two-tailed Student’s *T*-test. *p*-values < 0.05 were considered statistically significant.

## Results

### The effect of peanuts addition to diet on mice weight, food intake, and liver and adipose tissue weight

**Figure 1:**
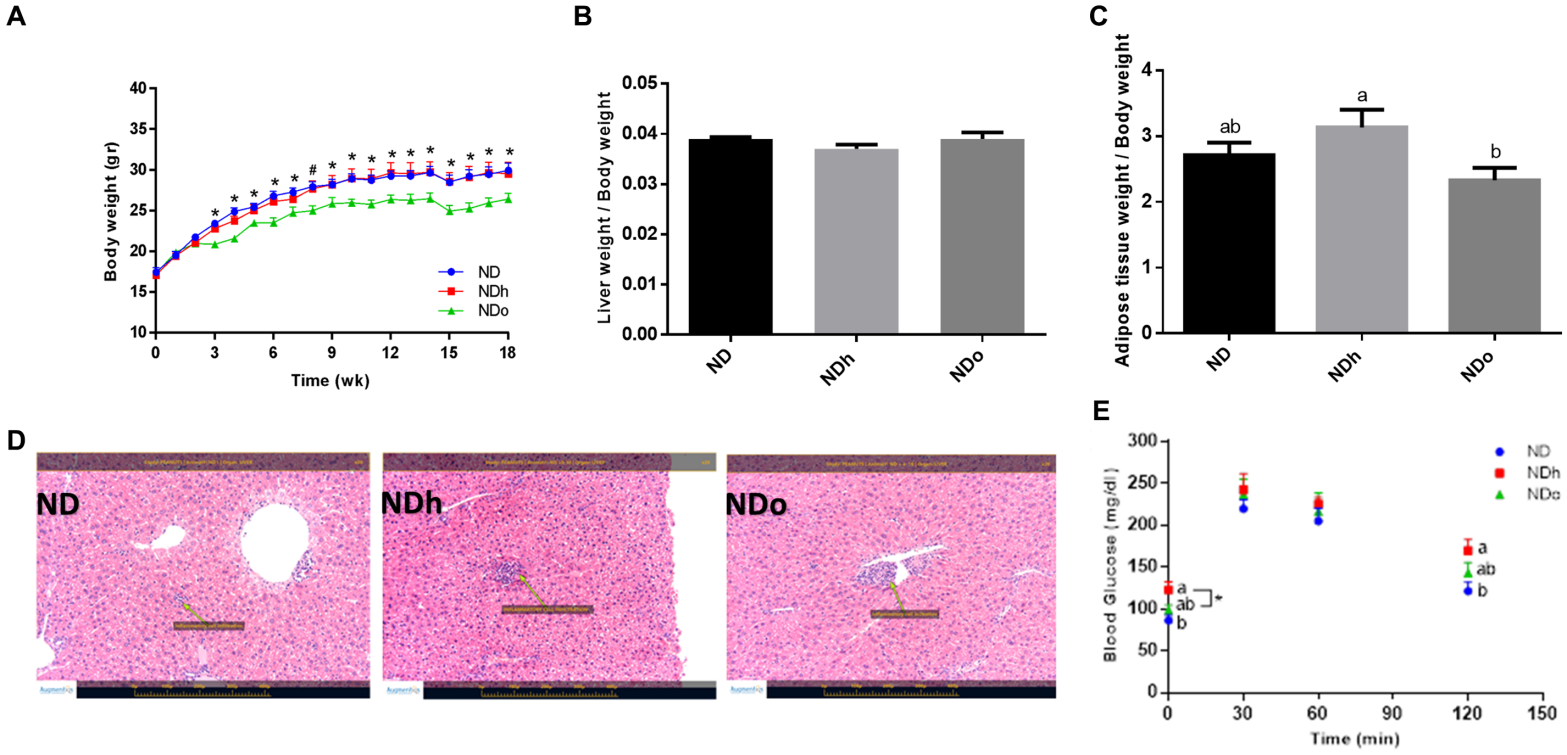
The effect of diets on body, liver, and adipose tissue weight and glucose tolerance. Mice consumed either a normal diet (ND), a ND diet plus 4% (w/w) of Hanoch (NDh), or Hanoch-Oleic (NDo) peanuts for 18 weeks. Body weight **(A)**, liver weight to body weight **(B)**, and adipose tissue weight to body weight **(C)** ratios were measured. Representative liver H&E staining from ND, NDh, and NDo. Arrows indicate normal minor inflammatory cell infiltration in all groups **(D)**. An oral glucose tolerance test (OGTT) was performed two weeks before the end of the experimental period **(E)**. A Tukey–Kramer HSD *post hoc* test and the Student’s *T*-test were performed. The values presented are mean ± SE (*n* = 8). Different letters indicate statistical variance at a significance level of *p* < 0.05.

Starting the third week and throughout the entire duration of the experiment (18 weeks), mice of the NDo group exhibited a lower body weight compared to the other groups, which was associated with reduced food consumption (Figure 1A; Table 4). Liver and adipose tissue weights were similar in all groups at the end of the experiment (Table 4). Given the observed variation in overall body weight, liver, and adipose tissue weight were also assessed as the ratio between these tissues-to-overall body weight. As shown in Figures 1B,C, enriching the diet with high oleic-acid peanuts resulted in a significant decline in the peri-gonadal visceral adipose-to-body weight ratio compared to NDh, whereas no significant change was observed in the liver-to-body weight ratio. The lack of effect of peanuts addition on liver fat accumulation was verified by histology and biochemical examination. Consistent with liver tissue weight, both analyses did not demonstrate profound alterations in the livers of the different groups (Figure 1D). The histological examination further revealed normal and equal inflammatory cell infiltration, which is frequently and spontaneously present in liver tissue (23), thus excluding the existence of liver inflammation in both peanuts-fed groups.

**Table 4:** Initial and final body weight, food intake, and tissue weight.

Parameters	ND	NDh	NDo
Initial body weight (g)	17.47 ± 0.53	17.11 ± 0.47	17.24 ± 0.22
Final body weight (g)	29.97 ± 0.86^a^	29.53 ± 1.4^a^	26.44 ± 0.67^b^
Food intake (g/day/mouse)	3.78 ± 0.05^a^	3.54 ± 0.08^ab^	3.31 ± 0.08^b^
Liver weight (g)	1.08 ± 0.04	1.04 ± 0.05	1.02 ± 0.08
Adipose tissue weight (g)	0.76 ± 0.07	0.89 ± 0.10	0.61 ± 0.09

The mice consumed either a normal diet (ND), a normal diet plus 4% (w/w) of Hanoch (NDh), or Hanoch-Oleic (NDo) peanuts for 18 weeks. A Tukey–Kramer HSD *post hoc* test was performed. The values presented are mean ± SE (*n* = 8). Different letters indicate statistical variance at a significance level of *p* < 0.05.

### Blood biochemistry profile, HOMA-IR index, and the glycemic response following peanuts addition to diet

No change was observed in plasma triglycerides, total cholesterol, HDL cholesterol, and free fatty acids levels. Similarly, plasma levels of the liver enzymes AST, ALT, and ALP did not differ between the groups. While insulin concentrations were equivalent between the groups, the HOMA-IR index was greater in the NDh compared with the other groups (Table 5).

**Table 5:** The effect of diets on lipid profiles, free fatty acid blood levels, liver enzyme, insulin level, and HOMA-IR Index.

Parameters	ND	NDh	NDo
Triglycerides (mg/dL)	101.67 ± 9.89	98.57 ± 5.18	78.6 ± 8.44
Total Cholesterol (mg/dL)	124.5 ± 5.94	132 ± 11.41	122 ± 6.04
HDL Cholesterol (mg/dL)	99.77 ± 3.97	104.91 ± 9.42	99.33 ± 4.09
Free fatty acids (nmol/μL)	0.29 ± 0.01	0.27 ± 0.01	0.27 ± 0.01
AST (IU/L)	52.6 ± 5.18	48.83 ± 2.52	48.17 ± 3.51
ALT (IU/L)	31.2 ± 7.56	24.5 ± 3.57	26 ± 3.81
ALP (IU/L)	69.67 ± 4.18	78.86 ± 6.53	78.33 ± 3.4
Insulin (mmol/L)	6.97 ± 0.65	8.85 ± 0.71	7.76 ± 0.61
HOMA-IR Index	0.51 ± 0.07^b^	0.97 ± 0.09^a^	0.62 ± 0.1^b^

The mice consumed either a normal diet (ND), a normal diet plus 4% (w/w) of Hanoch (NDh), or Hanoch-Oleic (NDo) peanuts for 18 weeks. At the end of the experiment, plasma triglycerides, cholesterol, high-density lipoprotein-cholesterol (HDL-cholesterol), alkaline phosphatase (AST), glutamic-pyruvic transaminase (ALT), glutamic oxaloacetic transaminase (ALP), free fatty acid levels and insulin levels were measured in the plasma (*n* = 8). HOMA-IR index was calculated by using the formula: [Fasting insulin concentration (microU/mL) * Glucose concentration (mg/dL)/405]. A Tukey–Kramer HSD *post hoc* statistical test was performed. The values presented are mean ± SE (*n* = 8). Different letters indicate statistical variance at a significant level of *p* < 0.05.

The influence of diet composition on the glycemic response was also evaluated using an OGTT during the 16th week of the experiment. The groups differed at two-time points, the fasting state and after 120 min of the glucose load. During these two time points, blood glucose levels were slightly, but significantly, higher in the NDh group compared to the control group (Figure 1E).

### The effect of peanuts addition to diet on liver gluconeogenesis and lipid metabolism

**Figure 2:**
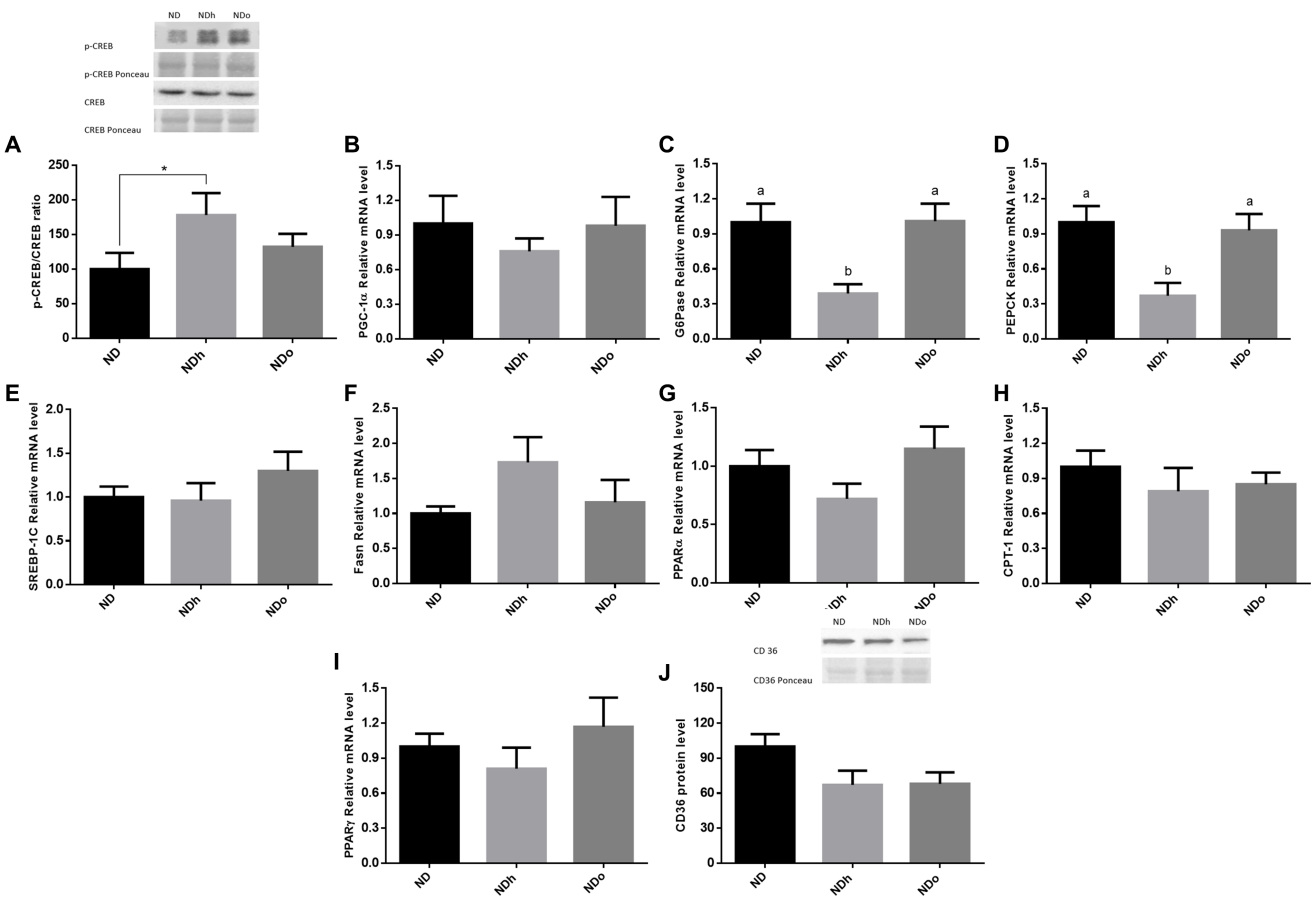
The effect of diets on key players that participate in glucose or lipid metabolism in the liver. Mice consumed either a normal diet (ND), a ND diet plus 4% (w/w) of Hanoch (NDh), or Hanoch-Oleic (NDo) peanuts for 18 weeks. The p-CREB/CREB protein ratio **(A)** was measured using Western blot where an unspecific band of total protein (Ponceau) was used as a control protein. PGC-1α **(B)**, G6Pase **(C)**, PEPCK **(D)** SREBP-1c **(E)**, Fasn **(F)**, PPARα **(G)**, CPT-1 **(H)**, PPARγ **(I)** gene expressions were measured at the transcription level (RT-PCR), and the results normalized to the 18S gene expression. CD36 protein levels **(J)** were measured using Western blots where an unspecific band of total protein (Ponceau) was used as a control protein. A Tukey’s Kramer *post hoc* test and Student’s *T*-test were performed. The values displayed are mean ± standard error. Columns marked with different letters indicate statistically significant variances at *p* < 0.05, *p < 0.05 vs. the NDo group.

To reveal the effect of peanuts on liver gluconeogenesis, the expression of key players that participate in this metabolic pathway was examined. The NDh diet led to a significant increase in the p-CREB (ser133)/CREB ratio compared to the ND group (Figure 2A). However, this change paralleled to a profound decrease in the expression of the main gluconeogenic enzymes, G6Pase and PEPCK, and a trend toward a decrease in the expression of the co-activator PGC-1α (Figures 2B–D). CREB phosphorylation ratio as well as G6Pase and PEPCK expression were unaffected in the NDo group. Concerning lipid metabolism, there were no significant differences between the experimental groups in the expression of SREBP-1c, Fasn, PPARα, CPT-1, and PPARγ nor CD36 protein levels (Figures 2E–J).

**Figure 3:**
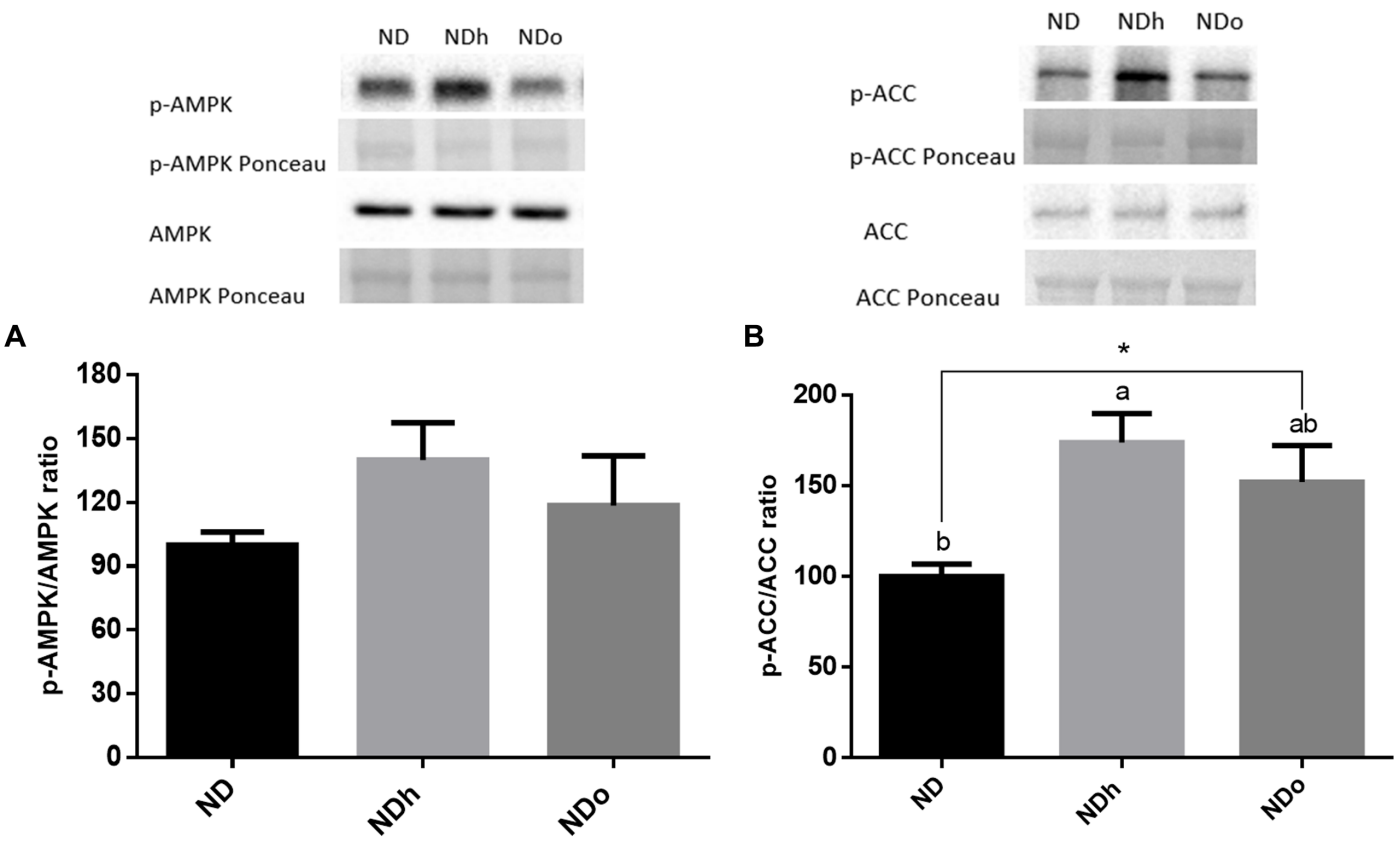
The effect of diets on key players that regulate glucose lipid metabolism in the liver. Mice consumed either a normal diet (ND), a ND diet plus 4% (w/w) of Hanoch (NDh), or Hanoch-Oleic (NDo) peanuts for 18 weeks. The p-AMPK/AMPK protein ratio **(A)**, and p-ACC/ACC protein ratio **(B)** were measured using Western blots where an unspecific band of total protein (Ponceau) was used as a control protein. A Tukey’s Kramer *post hoc* test and Student’s *T*-test were performed. The values displayed are mean ± standard error. Columns marked with different letters indicate statistically significant variances at *p* < 0.05, ^*^p < 0.05 vs. the ND group.

AMPK is an essential kinase known to orchestrate numerous signaling pathways that modulate carbohydrates and lipid metabolism. AMPK activation, as assessed by the ratio between α-subunit phosphorylation at Thr172 to total protein, was unaltered by peanuts addition to the diet (Figure 3A). However, ACC phosphorylation, a well-accepted representative of AMPK activity was significantly enhanced by the addition of regular peanuts to the diet while only a tendency toward a higher ratio was observed in the NDo group (Figure 3B).

### The effect of peanuts addition to diet on adipose tissue lipid metabolism

**Figure 4:**
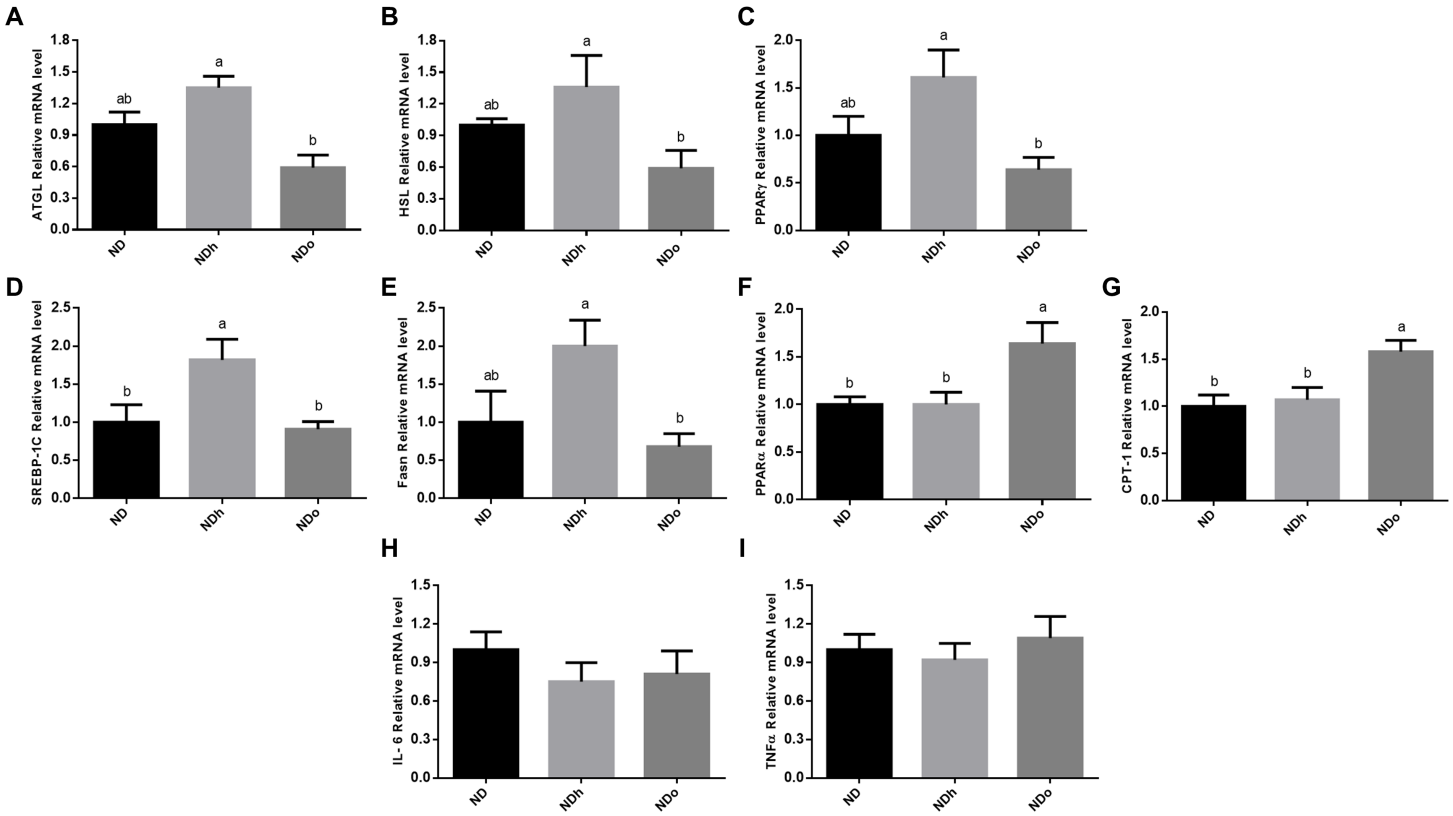
The effect of diets on key players that participate in lipid metabolism and inflammation in adipose tissue. Mice consumed either a normal diet (ND), a ND diet plus 4% (w/w) of Hanoch (NDh), or Hanoch-Oleic (NDo) peanuts for 18 weeks. The ATGL **(A)**, HSL **(B)**, PPARγ **(C)**, SREBP-1c **(D)**, Fasn **(E)**, PPARα **(F)**, CPT-1 **(G)**, Il-6 **(H)**, and TNFα **(I)** genes expressions were measured at the transcription level (RT-PCR), and the results normalized to the 18S gene expression. The values displayed are mean ± standard error. Columns marked with different letters indicate statistically significant variances at *p* < 0.05.

The effect of peanuts consumption on metabolic pathways related to lipids in adipose tissue was evaluated at the expression level. The expression of the two predominant enzymes that govern adipose tissue TG lipolysis, i.e., ATGL and HSL, was higher in the NDh group compared with NDo (Figures 4A,B). A similar pattern was observed in the expression of PPARγ, SREBP-1C, and FAS, which are key players in lipogenesis (Figures 4C–E). Finally, PPARα and CPT-1 mRNA levels were upregulated in the NDo compared to ND and NDh groups suggesting an elevated capacity to utilize lipids for energy within this tissue (Figures 4F,G). AMPK activation (measured by phospho-to-total AMPK ratio) and CD36 protein levels were unaffected by the addition of peanuts to the diet, nor by the peanut cultivar (data not shown).

### The effect of peanuts addition to diet on adipose tissue inflammation

The addition of peanuts to a normal diet did not elicit any effect on adipose tissue inflammation, as revealed by the expression levels of pro-inflammatory genes in this tissue (Figures 4H,I).

### The effect of peanuts addition to diet on intestinal microbiota composition

**Figure 5:**
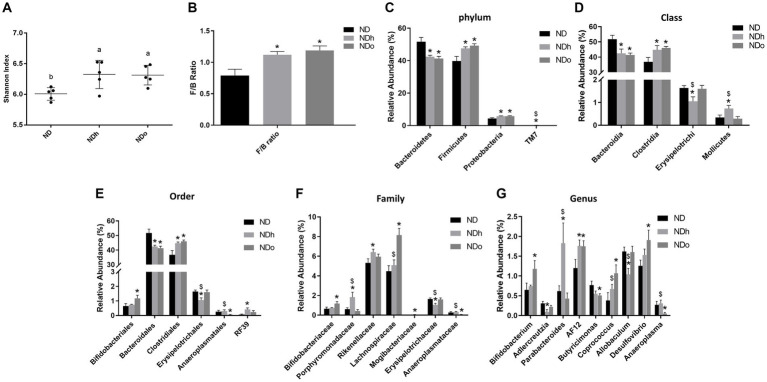
Shannon index **(A)**, F/B ratio **(C)**, and the relative abundance (%) of dominant phyla **(B)**, class **(D)**, order **(E)**, family **(F)**, and genus **(G)** in mice consumed either a normal diet (ND), a ND diet plus 4% (w/w) of Hanoch (NDh), or Hanoch-Oleic (NDo) peanuts for 18 weeks (*n* = 5). The values displayed are mean ± SE. ^*^p < 0.05 vs. ND group. $p < 0.05 vs. the NDo group.

**Figure 6:**
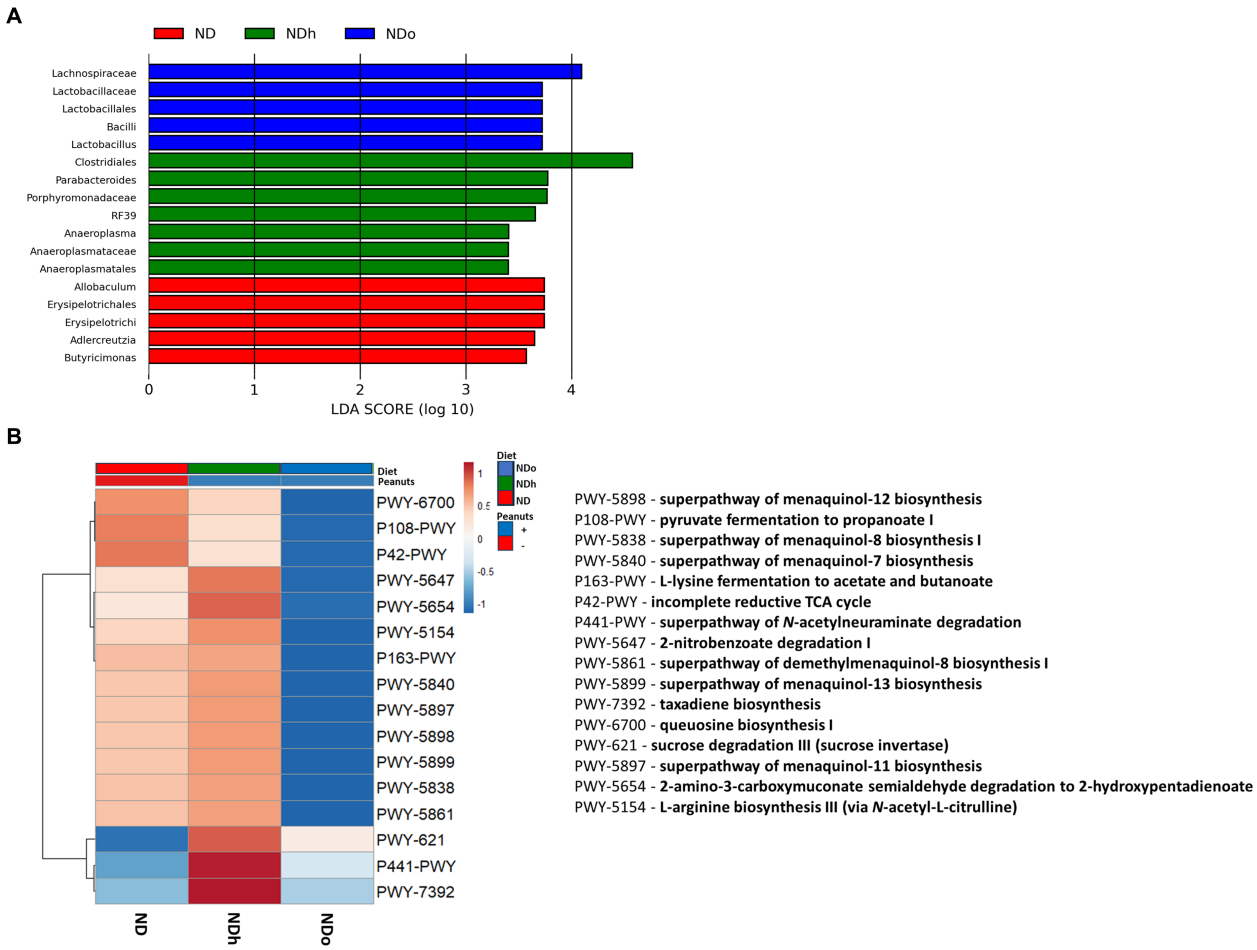
LEfSe analysis with LDA score > 3.0 **(A)** and functional profile predicted by PICRUSt **(B)** in mice consumed either a normal diet (ND), a ND diet plus 4% (w/w) of Hanoch (NDh), or Hanoch-Oleic (NDo) peanuts for 18 weeks.

The Shannon test, a measure of the α-diversity of the intestinal bacterial population, was higher in NDh and NDo groups compared to the control group (ND; Figure 5; Supplementary Table 1). Alterations at all taxonomic levels are elaborated in Figure 5; Supplementary Table 1. At the phyla level, the relative abundance of Proteobacteria, and Firmicutes was higher whereas that of Bacteroidetes was lower in the peanuts-added groups. Thus, the F/B ratio was also elevated in those groups. Only the NDh group exhibited an augmentation in TM7 phyla levels. At the class level, in contrast to Bacteroidia, Clostridia relative levels were more abundant in both NDh and NDo compared to control group an. The relative prevalence of Mollicutes was augmented while that of Erysipelotrichi was diminished in the NDh group. At the order level, high-oleic peanuts addition led to an increase and decrease in the amounts of Bifidobacteriales and Anaeroplasmatales, respectively. Conversely, the order of Erysipelotrichales was decreased while RF39 increased in NDh, At the family level, enhanced Bifidobacteriaceae, Lachnospiraceae along with lower Anaeroplasmataceae levels were noted in the NDo group. Whereas Erysipelotrichaceae levels were decreased, those of Porphyromonadaceae, Rikenellaceae, and Mogibacteriaceae were elevated in the NDh group. At the genus level, the relative abundance of *Bifidobacterium*, *Coprococcus*, and *Desulfovibrio* was enhanced while that of *Butyricimonas* and *Adlercreutzia* was diminished in the NDo group. In the NDh group, levels of the *Adlercreutzia* and *Allobaculum* genera were reduced, though concomitant increased *Parabacteroides* level was also registered in this group. The LDA Effect Size (LEfSE) was performed to find bacterial members that drive differences between groups (Figure 6).

The utilization of LEfSe analysis, using a threshold score of >3, uncovered variations in the abundance of several taxa, which are largely aligned with the previously mentioned changes in the relative abundance. Among the differences explicitly revealed by the LEfSe analysis was the enrichment of the *lactobacillus* genus, which was associated with the consumption of high-oleic peanuts (Figure 6). PICRUSt 2 was further applied to obtain functional analyses of the microbiota. PICRUSt-predicted functional profiles, based on MetaCyc metabolic pathways database, identified 16 pathways in which notable changes were found between groups. These metabolic pathways are elaborated in Figure 6.

## Discussion

The considerable impact of regular peanuts consumption on the physiological state of the individual is highlighted by data from epidemiologic and clinical studies. While much emphasis is placed on the phytochemical compounds found in peanuts, lipid composition is far from unnoticed, and variations in this component are thought to result in substantially different outcomes. The current, pioneer research describes the metabolic alterations brought on by a novel, high-oleic cultivar called “Hanoch-Oleic” under a normal, non-pathological setting. Findings revealed that consumption of the new, high-oleic peanuts cultivar had favorable metabolic effects on body weight and composition, insulin sensitivity, as well as significant positive microbial composition remodeling.

The high-oleic acid content in peanuts was associated with a slower rate of weight gain, most likely due to reduced food consumption. Several lines of evidence support the role of dietary oleic acid on food intake and feeding regulation in both, rodents and humans (24–26). Following absorption, dietary oleic acid can undergo diverse steps within duodenal and jejunal enterocytes to give rise to cellular oleoylethanolamide (OEA). This type of ethanolamide has been the subject of recent studies which demonstrated its anorexigenic properties, which are predominantly attributed to its ability to act as PPARα’s high-affinity agonist, among other proposed mechanisms (25, 26). Intestinal oleic acid may also be obtained through endogenous synthesis from stearic acid by the enzymatic activity of Stearoyl-CoA desaturase (also known as Δ-9-desaturase). Nonetheless, a high-oleic acid (low stearic acid) diet appears to be superior over high stearic acid (low oleic acid) diet in terms of increasing jejunal OEA levels along with greater satiation in mice. Thus, dietary oleic rather, than stearic acid, is advantageous for reducing food intake (27). Furthermore, the higher linoleic-oleic ratio of the Hanoch cultivar did not produce an equivocal result as oleic acid. Though inconsistency exists, this finding is in agreement with previous studies showing greater appetite suppression with the addition of oleic acid compared to linoleic acid (24, 28–31). Consistently, the anorexigenic effects of linoleoylethanolamide were suggested to be lesser than those mediated by OEA.

Results further indicate differences in fat distribution among peanuts-fed groups, with greater fat accumulation within peri-gonadal visceral fat in the NDh group, compared with the NDh group. These findings dovetail with the discrepancy in visceral adipose expression pattern between those groups, with that of the NDo group implying decreased lipolysis and lipogenesis on one hand and enhanced fatty acid oxidation on the other. Changes in insulin sensitivity were also observed in the traditional peanuts-fed group when compared to the other groups, as evidenced by higher fasting and 120-min-post OGTT blood glucose levels, as well as HOMA-IR measurements in the NDh group. Still, the hepatic metabolic state, as indicated by triglyceride contents and the expression of essential genes involved in key metabolic pathways, was not profoundly altered by peanuts consumption nor cultivar type. Distinctively, the expression of gluconeogenic genes was downregulated in the NDh group, presumably due to selective insulin resistance, with the gluconeogenesis pathway being less affected. Likewise, inflammatory markers in liver tissue were unaffected by diets. Thus, although the liver was less affected by the diet regimens, findings do imply some influence with high-oleic peanuts consumption eliciting more favorable metabolic outcomes compared to the traditional cultivar. These results stand in line with previous works conducted in animal models which infer preferable metabolic effects for high-oleic peanuts/peanut oil consumption in general and in comparison to the conventional cultivar (14, 31–34).

Since clinical data are currently scarce, the metabolic effects of high-oleic peanuts in humans are generally obscure and left to be disclosed. Body weight/composition changes are mixed, with some proposing that high-oleic peanuts promote advantageous, or less harmful, outcomes on these parameters. The influences of peanuts on diabetes and metabolic syndrome in humans have been assessed by two separate meta-analyses (35, 36). Overall, results refute the significant impact of peanuts intake on these pathologies. Yet, importantly, these meta-analyses were not designed to evaluate the effect of high-oleic peanuts in particular. An umbrella review reported recently the long-term benefits of nuts in general, and peanuts in specific, on the cardiovascular system. Although the underlying mechanism/s remain debatable, results indicate an enhanced beneficial effect on HDL levels by the high-oleic peanuts (1, 37). The present study, along with previously published investigations, encourages the conduction of clinical trials utilizing high-oleic peanuts to clarify and comprehend their specific significance.

After extended feeding on peanuts, discernible modifications in the composition of the gut microbiota were observed. Regardless of cultivar., peanut-fed animals were characterized by a greater F/B ratio, which was driven by concurrent increases and decreases in Firmicutes and Bacteroidetes abundance, respectively. Increased F/B ratio has long been employed as a characteristic of the dysbiosis associated with obesity and T2D, with the surmise that the former is a consequence of higher caloric availability that may accompany this altered proportion (38). Despite the popularity of this measure, evidence from human and animal models has cast doubt on its usage and forenamed interpretation (14, 39, 40). As a result, the consequences of the higher F/B ratio in the peanut-fed groups cannot be drawn at this time.

Unique adjustments were discovered in each group. In the NDh group, a considerable decrease in the Firmicutes class of Erysipelotrichi (and its consistent order and family of Erysipelotrichales and Erysipelotrichaceae, respectively) was noticed. Several liver-related metabolic perturbations have been suggested to emanate from the expansion of Erysipelotrichales. An overgrowth of Erysipelotrichales has been implicated in the pathophysiology/etiology of NASH in a mice model. It has also been posited that Erysipelotrichales increased levels may insinuate the futuristic development of cirrhosis on the background of NAFLD as well as liver steatosis under a choline-deficient diet in human subjects (Erysipelotrichi). Reducing Erysipelotrichaceae, however, resulted in the alleviation of parenteral-induced liver injury (41). Noteworthy, the differences presented here are accredited to disparities in the Allobaculum genus. Even though the Allobaculum genus was linked to desirable results, causality has not yet been established. Moreover, rebuts findings, of detrimental consequences, are claimed (42–46) and are corroborated by the detection of lower Allobaculum levels following bean consumption (47). Consequently, the importance of aberrant Allobaculum genus levels observed in this work after the consumption of traditional peanuts is currently unknown and needs to be ascertained.

The relative abundance of the class of Mollicutes was additionally increased in the NDh group and is attributed to the flourishment of the RF39 order, while levels of Anaeroplasmatales remained unaltered. Formerly, the Mollicutes class was associated with the establishment of obesity (48–50). At the same time, the RF39 family is potentially an acetate producer (47), which has been postulated to intensify the expression of hepatic genes that participate in fatty acid oxidation and hence negatively affects adiposity (51). In line with this, increased expansion of the acetate and propionate producers, Porphyromonadaceae (*Parabacteroides* genus) and Rikenellaceae families (52, 53), was registered in the NDh group, with the abundance of the former substantially outnumbering that of the high-oleic-fed group. In both humans and mice, these families were shown to be adversely linked with visceral fat mass and/or BMI (47, 53–60). Recently, the connection between *Parabacteroides* spp. and multiple pathologies was reviewed, with decreased levels being described in obesity, NAFLD, inflammatory bowel disease, and metabolic syndrome, among others. Given the positive effects ascribed to the *Parabacteroides spp*., these modifications may, speculatively, have a role in the pathophysiology of various diseases/conditions (61). Nevertheless, intervention studies are required for a conclusion to be made. In the current work, the NDo group exhibited no variation in Porphyromonadaceae enrichment, deducing that changes in the NDh group are due to lipid composition, i.e., higher n-6 linoleic acid contents. However, the profound ~3-fols increment of Porphyromonadaceae in the current study contradicts an earlier study that indicated a significant decrease in the presence of this family in mice whose food was supplemented with *n* − 6 (soy oil) (62). While the reason for this discrepancy, is unknown, it is possible that the inclusion of oleic along with linoleic acid may execute disparate outcomes of enhancement rather than inhibition in the thrive of Porphyromonadaceae. In summary, despite the less health-conductive metabolic effects described earlier for the NDh group, compositional alterations implemented by this traditional peanuts cultivar reflect the accommodation of favorable microbiota.

Alterations of gut microbiota composition following habitual consumption of high-oleic peanuts encompass a marked rise in the relative enrichment of the commensal genus *Bifidobacterium*. Compelling scientific evidence supports the health-promoting properties of Bifidobacterium, which is why it constitutes a frequently utilized probiotic (63, 64). Prebiotics are a dominant factor acknowledged to facilitate the bloom of bifidobacterial (64). Nevertheless, the lack of effect in the NDh group, stipulates lipid composition *per-se* imbued the perceived outcome. Results obtained by Zhao et al. advocate the capacity of high oleic-peanuts lipid composition to exert proliferative effects on Bifidobacterium. Indeed, in their work, high-oleic peanut oil successfully boosted the richness of this genus after 12 weeks of consumption (34). This raises the conjecture that adjustments of other taxa may facilitate the abundance of Bifidobacterium, secondary to the intricate interplay between them. Further research is warranted to determine the underlying mechanism/s. In the NDo group, the LDA score also identified differences in the abundance of *Lactobacillus*, another common probiotic bacteria with profound positive health-promoting effects on the host (65–67). This stands in line with previous findings indicating that oleic-acid promotes the survival of beneficial *lactobacilli* probiotics (68, 69).

There was also a noticeable decline in the lineage of the order Anaeroplasmatales. The level of the Mollicutes class, on the other hand, did not alter, perhaps due to the propensity of the RF39 levels to rise. Several data link depression and other disorders with the Anaeroplasmatales-*Anaeroplasma* line (70, 71). While scarce evidence exists to date, the connection between regular peanuts intake and mental well-being has been posited. In a 6 month trial conducted in young adults, 25 g of peanuts and 32 g of peanut butter were associated with improved anxiety and memory functioning, respectively. Additionally, peanut polyphenols were shown to be favorably connected with memory function while having an inverse relationship with stress response, which included depression. The anti-depressive impact of peanut-derived feces short-chain fatty acids (SCFAs) was also hypothesized (72). It would be intriguing to compare the effect of high-oleic peanuts on metal functions to that of traditional peanuts.

Another modification emphasizing the potentially elevated impact of high-oleic peanuts on depression is the observed rise in the prosperity of the *Coprococcus* genus. Of the Lachnospiraceae family, a noteworthy enrichment of the *Coprococcus* genus was found in the NDo group, and may partly explain the increased abundance of this family. *Coprococcus* species are acknowledged as SCFAs producers bacteria which are considered to evoke health-promoting outcomes on metabolic as well as mental systems (73–82) Accordingly, the advantages of eating this form of peanuts are strengthened by the potential of high-oleic peanuts to encourage the growth of the *Coprococcus* genus. Interestingly, a favorable relationship between *Bifidobacterium* and the *Coprococcus* bloom was discovered herein. This is in agreement with an earlier work where an expansion of *Coprococcus* genus was discovered following the intake of *Bifidobacterium Infantis* as a probiotic. In fact, in that study, *Coprococcus* genus upregulation was suggested to contribute to the food-allergies protection elicited by probiotics (83). Among food allergens, peanuts are considered to generate the most severe allergy. Of the scanty comparative studies conducted so far, high-oleic peanuts arguably execute less allergic response, conjecturally in light of their lower linoleic acid or allergenic protein contents (33). It is very tempting to suggest differential gut microbiota composition driven by the supplementation of high-oleic peanuts to the diet may aid in the long run to less food allergy, and as such add another interesting link between these peanuts and food allergy.

Finally, the high-oleic peanuts-fed group had higher *Desulfovibrio* genus richness. *Desulfovibrio* are sulfate-reducing bacteria which give rise to H2S levels. Currently, the association and the direct implication of *Desulfovibrio* in diverse pathologies are inconsistent, with both favorable and detrimental effects have been delineated (84–88). Nonetheless, H2S has recently gained much attention as a “gasotransmitters” with indispensable physiological functions, particularly in relation to the liver under positive energy balance (89). More research is needed to comprehensively define the role of *Desulfovibrio* genus.

## Conclusion

The current study investigated the outcomes of habitual consumption of peanuts, both traditional and a novel high-oleic cultivar., on key metabolic crossroads and the implication of these diet regimens on gut microbial populations. Findings demonstrated that the inclusion of high-oleic peanuts to a balanced diet, rather than regular peanuts, yields better metabolic outcomes. Essentially, frequent high-oleic peanuts consumption was found to harness the establishment of a healthy microbiota, with great emphasis on *Bifidobacterium, Lactobacillus,* and *Coprococcus* genera. These interoceptive modifications essentially symbolize the beneficial influencing outcomes of high-oleic peanuts habitual intake and underscore the leverage of these peanuts over the traditional cultivar. More research is required to elucidate the metabolic and microbiota composition effects evoked by peanuts consumption in general, and by each cultivar in particular, and to comprehensively describe the differences in the impact on these parameters between regular and high-oleic cultivars as well as between processing methods.

## Data availability statement

The datasets presented in this study can be found in online repositories. The names of the repository/repositories and accession number(s) can be found in the article/Supplementary material. The 16S rRNA sequences of mice gut microbiome of mice fed with Hanoch (NDh) and mice fed with Hanoch-Oleic (NDo) were uploaded to the NCBI SRA archive under BioProject accession number PRJNA955155. The control diet (ND) was taken from previous work BioProject accession number PRJNA728821.

## Ethics statement

The animal study was reviewed and approved by the Institutional Animal Care Ethics Committee of the Hebrew University of Jerusalem.

## Author contributions

SA-C: original draft preparation. NT-S: performing experiments and acquiring data. NT-S, GZ, RH, NS, and AN: data processing and analyzing. ZM, RH, and GZ: providing support in the experiments. ZM and NT-S: conceptualization and designing. All authors contributed to the article and approved the submitted version.

## Funding

The authors declare that this study received funding from the Israeli Ground Nuts Production and Marketing Board. The funder was not involved in the study design, collection, analysis, interpretation of data, the writing of this article, or the decision to submit it for publication.

## Conflict of interest

The authors declare that the research was conducted in the absence of any commercial or financial relationships that could be construed as a potential conflict of interest.

## Publisher’s note

All claims expressed in this article are solely those of the authors and do not necessarily represent those of their affiliated organizations, or those of the publisher, the editors and the reviewers. Any product that may be evaluated in this article, or claim that may be made by its manufacturer, is not guaranteed or endorsed by the publisher.

## Footnotes

^1^http://huttenhower.sph.harvard.edu/galaxy

## References

[1] Balakrishna R Bjornerud T Bemanian M Aune D Fadnes LT. Consumption of Nuts and Seeds and Health Outcomes Including Cardiovascular Disease, Diabetes and Metabolic Disease, Cancer, and Mortality: An Umbrella Review. Adv Nutr. (2022) 13:2136–48. doi: 10.1093/advances/nmac077 36041171 PMC9776667

[2] Bonku R Yu J. Health aspects of peanuts as an outcome of its chemical composition. Food Sci Human Wellness. (2020) 9:21–30. doi: 10.1016/j.fshw.2019.12.005

[3] Çiftçi S Suna G. Functional components of peanuts (*Arachis Hypogaea* L.) and health benefits: A review. Future foods. (2022) 5:100140. doi: 10.1016/j.fufo.2022.100140

[4] Mora-Escobedo R Hernández-Luna P Joaquín-Torres IC Ortiz-Moreno A Robles-Ramirez MDC. Physicochemical properties and fatty acid profile of eight peanut varieties grown in Mexico. CyTA-J Food. (2015) 13:300–4. doi: 10.1080/19476337.2014.971345

[5] Settaluri V Kandala C Puppala N Sundaram J. Peanuts and their nutritional aspects—a review. Food Nutr Sci. (2012) 3:1644–50. doi: 10.4236/fns.2012.312215

[6] Bermudez B Lopez S Ortega A Varela LM Pacheco YM Abia R. Oleic acid in olive oil: from a metabolic framework toward a clinical perspective. Curr Pharm Des. (2011) 17:831–43. doi: 10.2174/138161211795428957, PMID: 21443481

[7] Palomer X Pizarro-Delgado J Barroso E Vazquez-Carrera M. Palmitic and Oleic Acid: The Yin and Yang of Fatty Acids in Type 2 Diabetes Mellitus. Trends Endocrinol Metab. (2018) 29:178–90. doi: 10.1016/j.tem.2017.11.009, PMID: 29290500

[8] Medeiros-de-Moraes IM Goncalves-de-Albuquerque CF Kurz ARM Oliveira FMJ de Abreu VHP Torres RC. Omega-9 Oleic Acid, the Main Compound of Olive Oil, Mitigates Inflammation during Experimental Sepsis. Oxidative Med Cell Longev. (2018) 2018:6053492. doi: 10.1155/2018/6053492 PMC6260523 30538802

[9] Fan Y Pedersen O. Gut microbiota in human metabolic health and disease. Nat Rev Microbiol. (2021) 19:55–71. doi: 10.1038/s41579-020-0433-9 32887946

[10] Shen TCD Pyrsopoulos N Rustgi VK. Microbiota and the liver. Liver Transpl. (2018) 24:539–50. doi: 10.1002/lt.25008 29316191

[11] Liu J-P Zou W-L Chen S-J Wei H-Y Yin Y-N Zou Y-Y. Effects of different diets on intestinal microbiota and nonalcoholic fatty liver disease development. World J Gastroenterol. (2016) 22:7353–64. doi: 10.3748/wjg.v22.i32.7353, PMID: 27621581 PMC4997650

[12] Saltzman ET Palacios T Thomsen M Vitetta L. Intestinal microbiome shifts, dysbiosis, inflammation, and non-alcoholic fatty liver disease. Front Microbiol. (2018) 9:61. doi: 10.3389/fmicb.2018.00061, PMID: 29441049 PMC5797576

[13] Grabherr F Grander C Effenberger M Adolph TE Tilg H. Gut dysfunction and non-alcoholic fatty liver disease. Front Endocrinol. (2019) 10:611. doi: 10.3389/fendo.2019.00611 PMC6742694 31555219

[14] Anavi-Cohen S Zandani G Tsybina-Shimshilashvili N Hovav R Sela N Nyska A. Metabolic and Microbiome Alterations Following the Enrichment of a High-Fat Diet With High Oleic Acid Peanuts Versus the Traditional Peanuts Cultivar in Mice. Front Nutr. (2022) 9:3756. doi: 10.3389/fnut.2022.823756, PMID: 35782916 PMC9240694

[15] Zandani G Anavi-Cohen S Sela N Nyska A Madar Z. Broccoli consumption attenuates inflammation and modulates gut microbiome composition and gut integrity-related factors in mice fed with a high-fat high-cholesterol diet. Food Nutr Res. (2021) 65:7631. doi: 10.29219/fnr.v65.7631

[16] Hall M Beiko RG. 16S rRNA gene analysis with QIIME2. Microbiome Analy. (2018) 1849:113–29. doi: 10.1007/978-1-4939-8728-3_8 30298251

[17] Callahan BJ McMurdie PJ Rosen MJ Han AW Johnson AJA Holmes SP. DADA2: High-resolution sample inference from Illumina amplicon data. Nat Methods. (2016) 13:581–3. doi: 10.1038/nmeth.3869, PMID: 27214047 PMC4927377

[18] McDonald D Price MN Goodrich J Nawrocki EP DeSantis TZ Probst A. An improved Greengenes taxonomy with explicit ranks for ecological and evolutionary analyses of bacteria and archaea. ISME J. (2012) 6:610–8. doi: 10.1038/ismej.2011.139, PMID: 22134646 PMC3280142

[19] Segata N Izard J Waldron L Gevers D Miropolsky L Garrett WS. Metagenomic biomarker discovery and explanation. Genome Biol. (2011) 12:1–18. doi: 10.1186/gb-2011-12-6-r60 PMC3218848 21702898

[20] Douglas GM Maffei VJ Zaneveld JR Yurgel SN Brown JR Taylor CM. PICRUSt2 for prediction of metagenome functions. Nat Biotechnol. (2020) 38:685–8. doi: 10.1038/s41587-020-0548-6, PMID: 32483366 PMC7365738

[21] Love MI Huber W Anders S. Moderated estimation of fold change and dispersion for RNA-seq data with DESeq2. Genome Biol. (2014) 15:1–21. doi: 10.1186/s13059-014-0550-8 PMC4302049 25516281

[22] Metsalu T Vilo J. ClustVis: a web tool for visualizing clustering of multivariate data using Principal Component Analysis and heatmap. Nucleic Acids Res. (2015) 43:W566–70. doi: 10.1093/nar/gkv468, PMID: 25969447 PMC4489295

[23] Thoolen B Maronpot RR Harada T Nyska A Rousseaux C Nolte T. Proliferative and nonproliferative lesions of the rat and mouse hepatobiliary system. Toxicol Pathol. (2010) 38:5S–81S. doi: 10.1177/0192623310386499, PMID: 21191096

[24] Tutunchi H Ostadrahimi A Saghafi-Asl M. The Effects of Diets Enriched in Monounsaturated Oleic Acid on the Management and Prevention of Obesity: a Systematic Review of Human Intervention Studies. Adv Nutr. (2020) 11:864–77. doi: 10.1093/advances/nmaa013, PMID: 32135008 PMC7360458

[25] Romano A Tempesta B Provensi G Passani MB Gaetani S. Central mechanisms mediating the hypophagic effects of oleoylethanolamide and N-acylphosphatidylethanolamines: different lipid signals? Front Pharmacol. (2015) 6:137. doi: 10.3389/fphar.2015.00137 26167152 PMC4481858

[26] Schwartz GJ Fu J Astarita G Li X Gaetani S Campolongo P. The lipid messenger OEA links dietary fat intake to satiety. Cell Metab. (2008) 8:281–8. doi: 10.1016/j.cmet.2008.08.005, PMID: 18840358 PMC2572640

[27] Igarashi M Iwasa K Hayakawa T Tsuduki T Kimura I Maruyama K. Dietary oleic acid contributes to the regulation of food intake through the synthesis of intestinal oleoylethanolamide. Front Endocrinol. (2022) 13:6116. doi: 10.3389/fendo.2022.1056116 PMC9886573 36733808

[28] Naughton SS Hanson ED Mathai ML McAinch AJ. The Acute Effect of Oleic-or Linoleic Acid-Containing Meals on Appetite and Metabolic Markers; A Pilot Study in Overweight or Obese Individuals. Nutrients. (2018) 10. doi: 10.3390/nu10101376, PMID: 30261617 PMC6213143

[29] Mennella I Savarese M Ferracane R Sacchi R Vitaglione P. Oleic acid content of a meal promotes oleoylethanolamide response and reduces subsequent energy intake in humans. Food Funct. (2015) 6:204–10. doi: 10.1039/c4fo00697f, PMID: 25347552

[30] Kamphuis MM Westerterp-Plantenga MS Saris WH. Fat-specific satiety in humans for fat high in linoleic acid vs fat high in oleic acid. Eur J Clin Nutr. (2001) 55:499–508. doi: 10.1038/sj.ejcn.1601222, PMID: 11423927

[31] Bimro ET Hovav R Nyska A Glazer TA Madar Z. High oleic peanuts improve parameters leading to fatty liver development and change the microbiota in mice intestine. Food Nutr Res. (2020) 64:4278. doi: 10.29219/fnr.v64.4278, PMID: 33033472 PMC7520627

[32] Vassiliou EK Gonzalez A Garcia C Tadros JH Chakraborty G Toney JH. Oleic acid and peanut oil high in oleic acid reverse the inhibitory effect of insulin production of the inflammatory cytokine TNF-α both in vitro and in vivo systems. Lipids Health Dis. (2009) 8:1–10. doi: 10.1186/1476-511X-8-25 19558671 PMC2706835

[33] Derbyshire EJ. A review of the nutritional composition, organoleptic characteristics and biological effects of the high oleic peanut. Int J Food Sci Nutr. (2014) 65:781–90. doi: 10.3109/09637486.2014.937799, PMID: 25017702

[34] Zhao Z Shi A Wang Q Zhou J. High Oleic Acid Peanut Oil and Extra Virgin Olive Oil Supplementation Attenuate Metabolic Syndrome in Rats by Modulating the Gut Microbiota. Nutrients. (2019) 11. doi: 10.3390/nu11123005, PMID: 31817909 PMC6950752

[35] Becerra-Tomas N Paz-Graniel I Hernandez-Alonso P Jenkins DJA Kendall CWC Sievenpiper JL. Nut consumption and type 2 diabetes risk: a systematic review and meta-analysis of observational studies. Am J Clin Nutr. (2021) 113:960–71. doi: 10.1093/ajcn/nqaa358 33471083

[36] Zhang Y Zhang D-Z. Relationship between nut consumption and metabolic syndrome: a meta-analysis of observational studies. J Am Coll Nutr. (2019) 38:499–505. doi: 10.1080/07315724.2018.1561341, PMID: 30716015

[37] Jafari Azad B Daneshzad E Azadbakht L. Peanut and cardiovascular disease risk factors: A systematic review and meta-analysis. Crit Rev Food Sci Nutr. (2020) 60:1123–40. doi: 10.1080/10408398.2018.1558395, PMID: 30638042

[38] Polidori I Marullo L Ialongo C Tomassetti F Colombo R di Gaudio F. Characterization of Gut Microbiota Composition in Type 2 Diabetes Patients: A Population-Based Study. Int J Environ Res Public Health. (2022) 19:15913. doi: 10.3390/ijerph192315913, PMID: 36497987 PMC9740005

[39] Magne F Gotteland M Gauthier L Zazueta A Pesoa S Navarrete P. The Firmicutes/Bacteroidetes Ratio: A Relevant Marker of Gut Dysbiosis in Obese Patients? Nutrients. (2020) 12:1474. doi: 10.3390/nu12051474, PMID: 32438689 PMC7285218

[40] Quesada-Vazquez S Aragones G Del Bas JM Escote X. Diet, Gut Microbiota and Non-Alcoholic Fatty Liver Disease: Three Parts of the Same Axis. Cells. (2020) 9:176. doi: 10.3390/cells9010176, PMID: 31936799 PMC7016763

[41] Carter JK Bhattacharya D Borgerding JN Fiel MI Faith JJ Friedman SL. Modeling dysbiosis of human NASH in mice: Loss of gut microbiome diversity and overgrowth of Erysipelotrichales. PLoS One. (2021) 16:e0244763. doi: 10.1371/journal.pone.0244763, PMID: 33395434 PMC7781477

[42] Zheng Z Lyu W Ren Y Li X Zhao S Yang H. Allobaculum Involves in the Modulation of Intestinal ANGPTLT4 Expression in Mice Treated by High-Fat Diet. Front Nutr. (2021) 8:690138. doi: 10.3389/fnut.2021.690138, PMID: 34095196 PMC8171929

[43] Ren Y Wu S Xia Y Huang J Ye J Xuan Z. Probiotic-fermented black tartary buckwheat alleviates hyperlipidemia and gut microbiota dysbiosis in rats fed with a high-fat diet. Food Funct. (2021) 12:6045–57. doi: 10.1039/D1FO00892G, PMID: 34037655

[44] Hernandez AR Watson C Federico QP Fletcher R Brotgandel A Buford TW. Twelve Months of Time-Restricted Feeding Improves Cognition and Alters Microbiome Composition Independent of Macronutrient Composition. Nutrients. (2022) 14:3977. doi: 10.3390/nu14193977, PMID: 36235630 PMC9572159

[45] Ding L Ren S Song Y Zang C Liu Y Guo H. Modulation of gut microbiota and fecal metabolites by corn silk among high-fat diet-induced hypercholesterolemia mice. Front Nutr. (2022) 9:935612. doi: 10.3389/fnut.2022.935612, PMID: 35978956 PMC9376456

[46] van Muijlwijk GH van Mierlo G Jansen PW Vermeulen M Bleumink-Pluym NM Palm NW. Identification of Allobaculum mucolyticum as a novel human intestinal mucin degrader. Gut Microbes. (2021) 13:1966278. doi: 10.1080/19490976.2021.1966278, PMID: 34455931 PMC8409761

[47] Lutsiv T McGinley JN Neil-McDonald ES Weir TL Foster MT Thompson HJ. Relandscaping the Gut Microbiota with a Whole Food: Dose-Response Effects to Common Bean. Foods. (2022) 11:1153. doi: 10.3390/foods11081153, PMID: 35454741 PMC9025344

[48] Turnbaugh PJ Backhed F Fulton L Gordon JI. Diet-induced obesity is linked to marked but reversible alterations in the mouse distal gut microbiome. Cell Host Microbe. (2008) 3:213–23. doi: 10.1016/j.chom.2008.02.015, PMID: 18407065 PMC3687783

[49] Zhang C Zhang M Pang X Zhao Y Wang L Zhao L. Structural resilience of the gut microbiota in adult mice under high-fat dietary perturbations. ISME J. (2012) 6:1848–57. doi: 10.1038/ismej.2012.27, PMID: 22495068 PMC3446802

[50] Clarke SF Murphy EF Nilaweera K Ross PR Shanahan F O'Toole PW. The gut microbiota and its relationship to diet and obesity: new insights. Gut Microbes. (2012) 3:186–202. doi: 10.4161/gmic.20168, PMID: 22572830 PMC3427212

[51] Kondo T Kishi M Fushimi T Kaga T. Acetic acid upregulates the expression of genes for fatty acid oxidation enzymes in liver to suppress body fat accumulation. J Agric Food Chem. (2009) 57:5982–6. doi: 10.1021/jf900470c, PMID: 19469536

[52] Lei Y Tang L Liu S Hu S Wu L Liu Y. Parabacteroides produces acetate to alleviate heparanase-exacerbated acute pancreatitis through reducing neutrophil infiltration. Microbiome. (2021) 9:115. doi: 10.1186/s40168-021-01065-2, PMID: 34016163 PMC8138927

[53] Tavella T Rampelli S Guidarelli G Bazzocchi A Gasperini C Pujos-Guillot E. Elevated gut microbiome abundance of Christensenellaceae, Porphyromonadaceae and Rikenellaceae is associated with reduced visceral adipose tissue and healthier metabolic profile in Italian elderly. Gut Microbes. (2021) 13:1–19. doi: 10.1080/19490976.2021.1880221, PMID: 33557667 PMC7889099

[54] Tamura M Hoshi C Kobori M Takahashi S Tomita J Nishimura M. Quercetin metabolism by fecal microbiota from healthy elderly human subjects. PLoS One. (2017) 12:e0188271. doi: 10.1371/journal.pone.0188271, PMID: 29176866 PMC5703521

[55] Wang K Liao M Zhou N Bao L Ma K Zheng Z. *Parabacteroides distasonis* Alleviates Obesity and Metabolic Dysfunctions via Production of Succinate and Secondary Bile Acids. Cell Rep. (2019) 26:222–235.e5. doi: 10.1016/j.celrep.2018.12.028, PMID: 30605678

[56] Li M Wang S Li Y Zhao M Kuang J Liang D. Gut microbiota-bile acid crosstalk contributes to the rebound weight gain after calorie restriction in mice. Nat Commun. (2022) 13:2060. doi: 10.1038/s41467-022-29589-7, PMID: 35440584 PMC9018700

[57] Volynets V Louis S Pretz D Lang L Ostaff MJ Wehkamp J. Intestinal Barrier Function and the Gut Microbiome Are Differentially Affected in Mice Fed a Western-Style Diet or Drinking Water Supplemented with Fructose. J Nutr. (2017) 147:770–80. doi: 10.3945/jn.116.242859 28356436

[58] Ye J Zhao Y Chen X Zhou H Yang Y Zhang X. Pu-erh tea ameliorates obesity and modulates gut microbiota in high fat diet fed mice. Food Res Int. (2021) 144:110360. doi: 10.1016/j.foodres.2021.110360 34053553

[59] Liu Y Gao Y Ma F Sun M Mu G Tuo Y. The ameliorative effect of *Lactobacillus plantarum* Y44 oral administration on inflammation and lipid metabolism in obese mice fed with a high fat diet. Food Funct. (2020) 11:5024–39. doi: 10.1039/D0FO00439A, PMID: 32530448

[60] Li A Wang N Li N Li B Yan F Song Y. Modulation effect of chenpi extract on gut microbiota in high-fat diet-induced obese C57BL/6 mice. J Food Biochem. (2021) 45:e13541. doi: 10.1111/jfbc.13541 33570789

[61] Cui Y Zhang L Wang X Yi Y Shan Y Liu B. Roles of intestinal Parabacteroides in human health and diseases. FEMS Microbiol Lett. (2022) 369:fnac072. doi: 10.1093/femsle/fnac072 35945336

[62] Liu T Hougen H Vollmer AC Hiebert SM. Gut bacteria profiles of *Mus musculus* at the phylum and family levels are influenced by saturation of dietary fatty acids. Anaerobe. (2012) 18:331–7. doi: 10.1016/j.anaerobe.2012.02.004, PMID: 22387300

[63] Arboleya S Watkins C Stanton C Ross RP. Gut Bifidobacteria Populations in Human Health and Aging. Front Microbiol. (2016) 7:1204. doi: 10.3389/fmicb.2016.01204 27594848 PMC4990546

[64] Hidalgo-Cantabrana C Delgado S Ruiz L Ruas-Madiedo P Sánchez B Margolles A. Bifidobacteria and Their Health-Promoting Effects. Microbiol. Spectr (2017). 5, 5.3–21. doi: 10.1128/microbiolspec.BAD-0010-2016 PMC11687494 28643627

[65] Dempsey E Corr SC. Lactobacillus spp. for gastrointestinal health: current and future perspectives. Front Immunol. (2022) 13:245. doi: 10.3389/fimmu.2022.840245, PMID: 35464397 PMC9019120

[66] Mu Q Tavella VJ Luo XM. Role of *Lactobacillus reuteri* in human health and diseases. Front Microbiol. (2018) 9:757. doi: 10.3389/fmicb.2018.00757, PMID: 29725324 PMC5917019

[67] Lai Y Chen S Luo P Li P Du B. Dietary supplementation of Bacillus sp. DU106 activates innate immunity and regulates intestinal microbiota in mice. J Funct Foods. (2020) 75:104247. doi: 10.1016/j.jff.2020.104247

[68] Corcoran B Stanton C Fitzgerald G Ross R. Growth of probiotic lactobacilli in the presence of oleic acid enhances subsequent survival in gastric juice. Microbiology. (2007) 153:291–9. doi: 10.1099/mic.0.28966-0, PMID: 17185558

[69] Reitermayer D Kafka TA Lenz CA Vogel RF. Interrelation between Tween and the membrane properties and high pressure tolerance of *Lactobacillus plantarum*. BMC Microbiol. (2018) 18:1–14. doi: 10.1186/s12866-018-1203-y 30001697 PMC6044075

[70] Chen Q He Z Zhuo Y Li S Yang W Hu L. Rubidium chloride modulated the fecal microbiota community in mice. BMC Microbiol. (2021) 21:46. doi: 10.1186/s12866-021-02095-4 33588762 PMC7885239

[71] Wang H Wang G Banerjee N Liang Y Du X Boor PJ. Aberrant Gut Microbiome Contributes to Intestinal Oxidative Stress, Barrier Dysfunction, Inflammation and Systemic Autoimmune Responses in MRL/lpr Mice. Front Immunol. (2021) 12:651191. doi: 10.3389/fimmu.2021.651191, PMID: 33912174 PMC8071869

[72] Parilli-Moser I Dominguez-Lopez I Trius-Soler M Castellvi M Bosch B Castro-Barquero S. Consumption of peanut products improves memory and stress response in healthy adults from the ARISTOTLE study: A 6-month randomized controlled trial. Clin Nutr. (2021) 40:5556–67. doi: 10.1016/j.clnu.2021.09.020, PMID: 34656952

[73] Cui J Ramesh G Wu M Jensen ET Crago O Bertoni AG. Butyrate-Producing Bacteria and Insulin Homeostasis: The Microbiome and Insulin Longitudinal Evaluation Study (MILES). Diabetes. (2022) 71:2438–46. doi: 10.2337/db22-0168, PMID: 35972231 PMC9630078

[74] Vijay A Astbury S Le Roy C Spector TD Valdes AM. The prebiotic effects of omega-3 fatty acid supplementation: A six-week randomised intervention trial. Gut Microbes. (2021) 13:1–11. doi: 10.1080/19490976.2020.1863133, PMID: 33382352 PMC7781624

[75] Fernández J Redondo-Blanco S Gutiérrez-del-Río I Miguélez EM Villar CJ Lombo F. Colon microbiota fermentation of dietary prebiotics towards short-chain fatty acids and their roles as anti-inflammatory and antitumour agents: A review. J Funct Foods. (2016) 25:511–22. doi: 10.1016/j.jff.2016.06.032

[76] Yang R Shan S Shi J Li H An N Li S. *Coprococcus eutactus*, a Potent Probiotic, Alleviates Colitis via Acetate-Mediated IgA Response and Microbiota Restoration. J Agric Food Chem. (2023) 71:3273–84. doi: 10.1021/acs.jafc.2c06697 36786768

[77] Nogal A Louca P Zhang X Wells PM Steves CJ Spector TD. Circulating Levels of the Short-Chain Fatty Acid Acetate Mediate the Effect of the Gut Microbiome on Visceral Fat. Front Microbiol. (2021) 12:711359. doi: 10.3389/fmicb.2021.711359, PMID: 34335546 PMC8320334

[78] Azcarate-Peril M Roach J Marsh A Chey WD Sandborn WJ Ritter AJ. A double-blind, 377-subject randomized study identifies Ruminococcus, Coprococcus, Christensenella, and Collinsella as long-term potential key players in the modulation of the gut microbiome of lactose intolerant individuals by galacto-oligosaccharides. Gut Microbes. (2021) 13:1957536. doi: 10.1080/19490976.2021.1957536, PMID: 34365905 PMC8354614

[79] Da Silva HE Teterina A Comelli EM Taibi A Arendt BM Fischer SE. Nonalcoholic fatty liver disease is associated with dysbiosis independent of body mass index and insulin resistance. Sci Rep. (2018) 8:1466. doi: 10.1038/s41598-018-19753-9, PMID: 29362454 PMC5780381

[80] Liang D Zhang L Chen H Zhang H Hu H Dai X. Potato resistant starch inhibits diet-induced obesity by modifying the composition of intestinal microbiota and their metabolites in obese mice. Int J Biol Macromol. (2021) 180:458–69. doi: 10.1016/j.ijbiomac.2021.02.209, PMID: 33711371

[81] Altemani F Barrett HL Gomez-Arango L Josh P McIntyre HD Callaway LK. Pregnant women who develop preeclampsia have lower abundance of the butyrate-producer Coprococcus in their gut microbiota. Pregn Hypertens. (2021) 23:211–9. doi: 10.1016/j.preghy.2021.01.002, PMID: 33530034

[82] Järbrink-Sehgal E Andreasson A. The gut microbiota and mental health in adults. Curr Opin Neurobiol. (2020) 62:102–14. doi: 10.1016/j.conb.2020.01.016 32163822

[83] Yang B Xiao L Liu S Liu X Luo Y Ji Q. Exploration of the effect of probiotics supplementation on intestinal microbiota of food allergic mice. Am J Transl Res. (2017) 9:376–85. PMID: 28337267 PMC5340674

[84] Chen L Gao Y Zhao Y Yang G Wang C Zhao Z. Chondroitin sulfate stimulates the secretion of H(2)S by Desulfovibrio to improve insulin sensitivity in NAFLD mice. Int J Biol Macromol. (2022) 213:631–8. doi: 10.1016/j.ijbiomac.2022.05.195, PMID: 35667460

[85] Chen Y-R Jing Q-L Chen F-L Zheng H Chen L-D Yang Z-C. Desulfovibrio is not always associated with adverse health effects in the Guangdong Gut Microbiome Project. PeerJ. (2021) 9:e12033. doi: 10.7717/peerj.12033 34466295 PMC8380029

[86] Hong Y Sheng L Zhong J Tao X Zhu W Ma J. *Desulfovibrio vulgaris*, a potent acetic acid-producing bacterium, attenuates nonalcoholic fatty liver disease in mice. Gut Microbes. (2021) 13:1–20. doi: 10.1080/19490976.2021.1930874, PMID: 34125646 PMC8205104

[87] Lin Y-C Lin H-F Wu C-C Chen C-L Ni Y-H. Pathogenic effects of Desulfovibrio in the gut on fatty liver in diet-induced obese mice and children with obesity. J Gastroenterol. (2022) 57:913–25. doi: 10.1007/s00535-022-01909-0, PMID: 35976494

[88] Lu G Zhang Y Ren Y Shi J-S Xu Z-H Geng Y. Diversity and Comparison of Intestinal Desulfovibrio in Patients with Liver Cirrhosis and Healthy People. Microorganisms. (2023) 11:276. doi: 10.3390/microorganisms11020276, PMID: 36838242 PMC9960842

[89] Li X Jiang K Ruan Y Zhao S Zhao Y He Y. Hydrogen Sulfide and Its Donors: Keys to Unlock the Chains of Nonalcoholic Fatty Liver Disease. Int J Mol Sci. (2022) 23:12202. doi: 10.3390/ijms232012202, PMID: 36293058 PMC9603526

