# Supplementary material for: Effects of high oleic acid peanuts on mice’s liver and adipose tissue metabolic parameters and gut microbiota composition
Source: Front Nutr. 2023 Jul 27;10:1205377. doi: 10.3389/fnut.2023.1205377 (PMC10415107; doi:10.3389/fnut.2023.1205377)
Supplement: Supplementary file 1 [file Table_1.docx]

**Supplementary Table 1.** Taxonomical levels and diversity indexes

|  | **ND** | **NDh** | **NDo** |
| --- | --- | --- | --- |
| **α diversity** | | | |
| Observed OUT’s | 141.80±3.20 | 155.80±9.64 | 153.00±4.53 |
| Shannon index | 6.00±0.07 | 6.32±0.11* | 6.31±0.08* |
| Pielou's index | 0.84±0.01 | 0.87±0.01* | 0.87±0.01* |
| **Phylum level** | | | |
| Bacteroidetes | 51.70±2.69 | 42.49±0.89* | 41.36±1.33* |
| Firmicutes | 39.75±2.86 | 47.50±1.01* | 49.20±1.20* |
| Proteobacteria | 4.36±0.52 | 5.75±0.28* | 5.80±0.27* |
| TM7 | 0.08±0.03 | 0.16±0.07*^,$^ | 0.11±0.03 |
| F/B ratio | 0.79±0.10 | 1.12±0.05* | 1.19±0.07* |
| **Class level** | | | |
| Bacteroidia | 51.69±2.69 | 42.49±2.82* | 41.36±1.33* |
| Clostridia | 36.84±2.93 | 44.72±2.80* | 46.05±0.93* |
| Erysipelotrichi | 1.63±0.11 | 1.05±0.20*^,$^ | 1.60±0.15 |
| Mollicutes | 0.34±0.11 | 0.73±0.14*^,$^ | 0.29±0.09 |
| **Order level** | | | |
| Bifidobacteriales | 0.65±0.17 | 0.74±0.03 | 1.18±0.21* |
| Bacteroidales | 51.69±2.69 | 42.49±0.89* | 41.36±1.33* |
| Clostridiales | 36.84±2.93 | 44.72±0.88* | 46.04±0.92* |
| Erysipelotrichales | 1.64±0.11 | 1.06±0.15*^,$^ | 1.60±0.15 |
| Anaeroplasmatales | 0.27±0.09 | 0.31±0.08^$^ | 0.05±0.03* |
| RF39 | 0.07±0.03 | 0.42±0.10* | 0.24±0.09 |
| **Family level** | | | |
| Bifidobacteriaceae | 0.65±0.17 | 0.74±0.03 | 1.18±0.21* |
| Porphyromonadaceae | 0.62±0.13 | 1.83±0.51*^,$^ | 0.43±0.14 |
| Rikenellaceae | 5.32±0.42 | 6.41±0.31* | 5.95±0.26 |
| Lachnospiraceae | 4.47±0.57 | 5.08±0.55^$^ | 8.15±0.69* |
| Mogibacteriaceae | 0.02±0.01 | 0.06±0.01* | 0.03±0.01 |
| Erysipelotrichaceae | 1.64±0.11 | 1.06±0.15*^,$^ | 1.60±0.15 |
| Anaeroplasmataceae | 0.27±0.09 | 0.31±0.08^$^ | 0.05±0.03* |
| **Genus level** |  |  |  |
| Bifidobacterium | 0.65±0.17 | 0.74±0.03 | 1.18±0.21* |
| Adlercreutzia | 0.31±0.05 | 0.09±0.06* | 0.22±0.04 |
| Parabacteroides | 0.62±0.13 | 1.83±0.51*^,$^ | 0.43±0.14 |
| AF12 | 1.20±0.22 | 1.76±0.15* | 1.75±0.14* |
| Butyricimonas | 0.77±0.10 | 0.55±0.07 | 0.51±0.04* |
| Coprococcus | 0.38±0.20 | 0.67±0.12^$^ | 1.07±0.21* |
| Allobaculum | 1.62±0.11 | 1.04±0.15*^,$^ | 1.60±0.15 |
| Desulfovibrio | 1.26±0.14 | 1.53±0.15 | 1.91±0.25* |
| Anaeroplasma | 0.27±0.09 | 0.31±0.08^$^ | 0.05±0.03* |

**Supplementary Table 1.** The effect of diets on the microbiota composition. Mice consumed either a normal diet (ND), a normal diet plus 4% (w/w) of **Hanoch (NDh), or Hanoch-Oleic (NDo)** peanuts for 18 weeks. Alpha- α-diversity parameters were calculated. The bacterial population was determined at phylum, class, order, family, and genus levels (n=5). The values displayed are mean ± SE. *p<0.05 versus ND group. ^$^p<0.05 versus the NDo group.
