# Supplementary figures and images for: Effects of high oleic acid peanuts on mice’s liver and adipose tissue metabolic parameters and gut microbiota composition
Source: Front Nutr. 2023 Jul 27;10:1205377. doi: 10.3389/fnut.2023.1205377 (PMC10415107; doi:10.3389/fnut.2023.1205377)

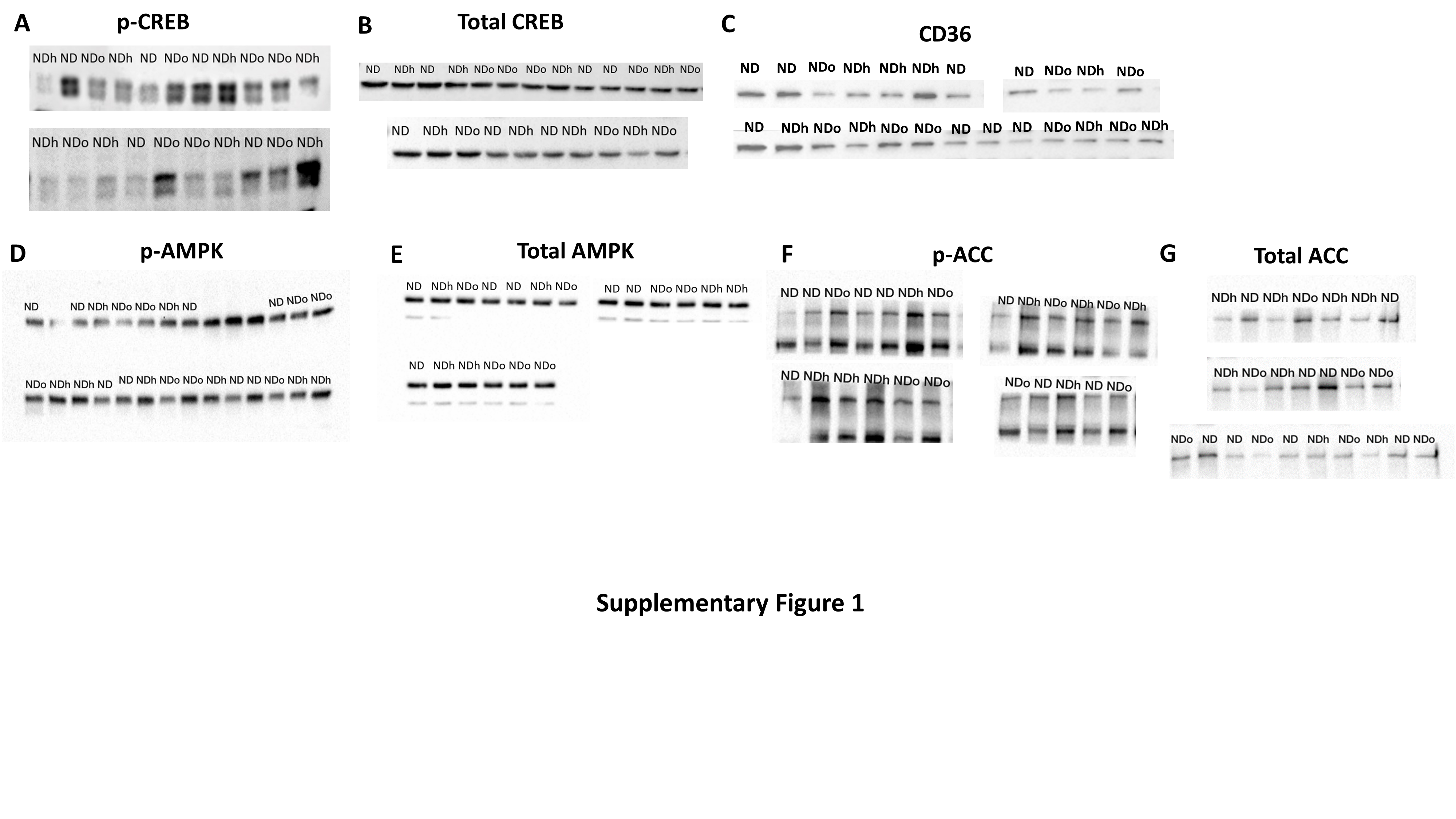

Supplement: Supplementary file 2 [file Image_1.tif]
